# Toward Personalized Oral Diagnosis: Distinct Microbiome Clusters in Periodontitis Biofilms

**DOI:** 10.3389/fcimb.2021.747814

**Published:** 2021-12-22

**Authors:** Roland Wirth, Bernadett Pap, Gergely Maróti, Péter Vályi, Laura Komlósi, Nikolett Barta, Orsolya Strang, János Minárovits, Kornél L. Kovács

**Affiliations:** ^1^ Department of Biotechnology, University of Szeged, Szeged, Hungary; ^2^ Biological Research Center, Institute of Plant Biology, Szeged, Hungary; ^3^ Department of Periodontology, University of Szeged, Szeged, Hungary; ^4^ Department of Oral Surgery, University of Szeged, Szeged, Hungary; ^5^ Department of Oral Biology and Experimental Dental Research, University of Szeged, Szeged, Hungary

**Keywords:** periodontitis, microbiome, read-based metagenome, 16S rRNA gene, genome-based metagenome, whole-genome sequencing, paper-point sampling, curette scaling

## Abstract

Periodontitis is caused by pathogenic subgingival microbial biofilm development and dysbiotic interactions between host and hosted microbes. A thorough characterization of the subgingival biofilms by deep amplicon sequencing of 121 individual periodontitis pockets of nine patients and whole metagenomic analysis of the saliva microbial community of the same subjects were carried out. Two biofilm sampling methods yielded similar microbial compositions. Taxonomic mapping of all biofilms revealed three distinct microbial clusters. Two clinical diagnostic parameters, probing pocket depth (PPD) and clinical attachment level (CAL), correlated with the cluster mapping. The dysbiotic microbiomes were less diverse than the apparently healthy ones of the same subjects. The most abundant periodontal pathogens were also present in the saliva, although in different representations. The single abundant species *Tannerella forsythia* was found in the diseased pockets in about 16–17-fold in excess relative to the clinically healthy sulcus, making it suitable as an indicator of periodontitis biofilms. The discrete microbial communities indicate strong selection by the host immune system and allow the design of targeted antibiotic treatment selective against the main periodontal pathogen(s) in the individual patients.

## 1 Introduction

The oral cavity is an open system, where the microbial community can survive in sessile biofilms under the constant fluctuation of materials and environmental stimuli. These highly organized and structured assemblies develop beneficial or harmful interactions with the host. Oral biofilms are easily accessible and excellent models to study biofilm lifestyles *in vivo* (Jia et al., 2018; Jakubovics and Shi, 2020). Dysbiotic oral microbial communities develop periodontal diseases (PDs), which comprise the mild (gingivitis) and severe (periodontitis) forms of gingival inflammation (Caton et al., 2018). Gingivitis is reversible, but periodontitis causes irreversible destruction of the gum and alveolar bone. PD is one of the most common diseases of mankind nowadays; about 20%–50% of the human population suffers from one or more forms of PD (Nazir, 2017; Lu et al., 2019; Kumar et al., 2020). Although PD is considered non-communicable and rarely a life-threatening condition, the multiple tooth losses and masticatory dysfunctions cruelly affect the quality of life and self-esteem, thereby imposing huge health care costs. Severe periodontitis is the sixth most prevalent disease worldwide, and almost 1 billion people are affected (Tonetti et al., 2017). It has been recognized that PD, and particularly periodontitis, is associated with a growing number of health complications and diseases in various parts of the human body, recently reviewed by Teles et al. (2021). There is accumulating evidence that communication between the microbiota and host is a two-way traffic (Harvey, 2017; Nędzi-Góra et al., 2017; Verma et al., 2018; Feres et al., 2021), although the temporal relationship between the dysbiotic microbiome and the periodontal inflammation is still a matter of debate (Van Dyke et al., 2020; Cugini et al., 2021).

Not surprisingly, the healthy and disturbed oral microbiota has been the subject of many studies from the classical discoveries of van Leeuwenhoek (Lane, 2015) to the introduction to more sophisticated microbiological cultivation (Sizova et al., 2012) and molecular biology tools (Socransky and Haffajee, 2005; Zaura et al., 2009; Zaura et al., 2021).

The relationship between oral and gut microbiota and the overall health status is multifarious, therefore having a precise picture of the oral microbiota is imperative for adequate diagnosis and therapy of numerous health-threatening systemic disorders. The treatment and prevention of periodontitis are difficult because of its complex etiology. Modifiable and non-modifiable risk factors have been recognized, which may lead to periodontal inflammation (Nazir, 2017; Kumar et al., 2020; Van Dyke et al., 2020).

Sample collection from the oral cavity is relatively easy. Sampling oral microbial biofilms is done using paper-point, oral swab, or curette techniques (Beikler et al., 2006; Persson et al., 2008; Teles et al., 2008; Santigli et al., 2017; Zaura et al., 2021). These methods allow targeted sampling, although the various tools may scoop out various depths of the biofilm. In addition, these devices furnish only a small amount of oral bacteria for further investigations. Alternatively, one can collect saliva samples of the subjects. This may yield a sufficient amount of microbial biomass, but saliva contains habitually the planktonic members of the oral microbiota hence cannot give precise information about the microbes harboring sessile biofilms in periodontal pockets (Belstrøm, 2020). One of the goals in the current study was to determine the effects of the various sampling methods on the composition of the microbial community relative to clinically healthy sulcus of the same subjects.

The technical limitations associated with small samples include the need for 16S rRNA amplicon sequencing of the polymerase chain reaction (PCR)-amplified DNA and the inherently present potential PCR bias, which may disturb the results (e.g., Pinto and Raskin, 2012). In addition, the targeted 16S rRNA gene may be present in the bacteria in multiple copies, which introduces a systematic error in the calculation of relative abundances in amplicon sequencing (Louca et al., 2018).

In order to obtain a more accurate picture of the biofilm microbiota, in this study, the 16S rRNA gene read-based amplicon sequences of samples taken from the periodontal pockets using both paper-point and curette techniques with genome-based metagenomes of the saliva collected from the same patients were compared. The individual variations among the subjects were studied by using both sampling techniques from four to eight separate periodontal pockets and one control paper-point sample of each patient.

## 2 Materials and Methods

### 2.1 Clinical Examination and Sampling

Only patients with probing pocket depth (PPD) of ≥4 mm in case of at least two pockets per quadrant were included in this study.

The exclusion criteria included periodontal treatment (other than supragingival cleaning), antibiotic or anti-inflammatory drug administration within the previous 6 months, acute infection of the oral cavity, known systemic diseases, pregnancy, smoking and drinking habits, and physical or mental disability to interfere with proper individual oral hygiene.

The study protocol was approved by the Institutional Review Board of the University of Szeged, Szeged, Hungary. A signed informed consent was obtained from each participant enrolled into the study at the Department of Periodontology, University of Szeged, Hungary. The diagnosis was established according to the guidelines of Chapple et al. (2018) and Tonetti et al. (2018).

#### 2.1.1 Periodontal Parameters

Prior to sample collection in 2017–2018, clinical parameters were registered (**Supplementary Material 1**), and microbiological samples were taken from at least two sites per quadrant. A single calibrated examiner (intra-examiner reliability was 95% within ±1 mm) carried out the clinical examinations. The examiner was calibrated through multiple repeated measurements of probing pocket depth (PPD), clinical attachment level (CAL), bleeding on probing (BoP), and Plaque Index (PI) in at least four teeth on five patients. Probing pocket depth (PPD) was measured with CP-15 (Hu-Friedy, Chicago, IL, USA) periodontal probes. The mean pocket depth of the sites sampled was 5.5 mm (range: 4–12 mm). Probing pocket depth (PPD), gingival recession, and CAL was measured with CP-15 (Hu-Friedy, Chicago, IL, USA) periodontal probes. Inflammation of gingiva was determined by Gingival Index (Löe, 1967). Evaluation of individual oral hygiene was carried out by PI (Silness and Löe, 1964). The PPD measures were categorized into three type groups (PPDT), i.e., clinically healthy sulcus (PPDT-1, PPD = 0–3 mm), shallow pocket (PPDT-2, PPD = 4–5 mm), and deep pocket (PPDT-3, PPD >5 mm). The mean CAL at the diseased teeth was 5.91 mm (SD: 2.04 mm), whereas the mean CAL at the clinically healthy gingival sulcus samples was 2.89 mm (SD: 1.54 mm); the difference between the sample groups was significant (p = 0.0001). The mean PI (Silness and Löe, 1964) values ranged from 0.66 to 0.5 for the diseased and healthy control samples, respectively. The mean Gingival Index (Löe, 1967) of the diseased teeth was 1.53 vs. 1.40 of healthy control teeth.

#### 2.1.2 Microbial Sampling

First, an unstimulated saliva sample was collected into a sterile plastic tube from each subject using a simple drooling method as in our previous studies (Wirth et al., 2020; Wirth et al., 2021). The samples were stored at -80°C until DNA isolation. Next, microbial samples were taken from periodontal pockets using sterile paper points (ISO50) from each subject. At least four periodontal pockets were sampled per individual. In addition, a control microbial sample was also taken from each subject, using a paper point, from the crevicular fluid of a single clinically healthy gingival sulcus. Finally, subgingival biofilm (plaque) samples (4–6 samples per individual) were taken with a sterile Langer curette (LC) from the surface of the affected teeth during the therapeutic elimination of the plaques. A separate LC was used for each tooth, and all collected biofilm samples were placed in individual sterile tubes for further analysis. The biofilms were retrieved from the curette by individual sterile paper points (size 70). The samples were stored at -80°C in sterile plastic tubes until DNA isolation.

### 2.2 DNA Isolation From Saliva and Subgingival Plaque Samples

Saliva samples were thawed, and 3 ml of each was centrifuged at 13,000 rpm for 5 min. Subgingival plaque samples taken by sterile paper points or LC were resuspended in 500 µl TE buffer (10 mM Tris, 1 mM EDTA, pH 8) and were also pelleted at 13,000 rpm for 5 min. DNA extractions were carried out from both sample types by using the Macherey-Nagel (Düren, Germany) NucleoSpin Soil DNA kit (Macherey-Nagel: 740780.250). Sample preparation and quality estimation were performed according to Wirth et al. (2021).

### 2.3 Next-Generation Sequencing of Subgingival Biofilm and Saliva Samples

The amplification, purification, and sequencing of the prokaryotic hypervariable V3-V4 region of 16S rRNA gene were performed as described in “Preparing 16S Ribosomal RNA Gene Amplicons for the Illumina MiSeq System” standard protocol provided by the supplier (Illumina). Prokaryotic 16S rRNA gene amplification, purification, and sequencing were performed as described in the standard protocol of the supplier (Illumina, San Diego, CA, USA). Briefly, the hypervariable V3-V4 region of the 16S rRNA gene was PCR-amplified, and DNA sequencing was carried out on an Illumina MiSeq machine using V2 sequencing chemistry (MiSeq Reagent Kit v2) (500 cycles). Detailed description of the applied method can be found in our previous article (Wirth et al., 2021).

#### 2.3.1 Amplicon Sequence Analysis of Subgingival Biofilm Samples

Amplicon sequencing data were handled in house-developed bioinformatics pipeline, containing five modules (Wirth et al., 2021). 0pt?>1) Sequencing preparation: join paired end fastq reads ($cat). 2) Trimming: raw sequences were trimmed by Trimmomatic (v.0.36.5: slidingwindow: 4:20, minlen: 200, leading: 3, trailing: 3) and checked with FastQC (v.0.11.8) (Bolger et al., 2014). 3) Taxonomic annotation: amplicon sequences were annotated with Kraken2 (v.2.0.8) using the NCBI RefSeq (genome) and RDP (16S rDNA amplicon) databases. 4) Filtration and normalization: Kraken feature table outputs were filtered by Kraken2 (–confidence 0.95) (Wood et al., 2019). Copy number normalization was done through the rrnDB (v.5.6) (Roller et al., 2016) database. MetagenomeSeq (v.1.16.0) was used to create normalized and scaled output of microbial abundances (–rel 0.1, –scale 1,000) (Paulson et al., 2013). 5) Statistics and visualization: Megan6 (v.6.18.1) was used to export data for statistical calculation and rarefaction estimation (Huson et al., 2016). The core composition of microbial taxa and the distribution of the top 10 most frequent microbes between the three types of samples (Paper-point healthy; Paper-point disease; and LC disease) were presented in Krona (v.2.6.1) and Circos (v.0.36.9) (Connors et al., 2009; Ondov et al., 2011). MetaCoMET (Metagenomics Core Microbiome Exploration Tool) was used for microbial core (composition 0.8, persistence 0.8) calculation (Wang et al., 2016). Alpha diversity was calculated with Shannon statistical method, and for principal component analysis, R program was employed (microeco package). Differences in alpha diversity and principal component analysis were calculated by Wilcoxon and PERMANOVA tests, respectively. Significantly different taxa were identified by STAMP (Statistical Analysis of Metagenomic Profiles) [two-sided t-test with 0.95 confidence intervals (p ≤ 0.05)] (Parks and Beiko, 2010) (**Supplementary Materials 2**, **3**). Minimum difference between proportions was set to 0.3, and Benjamini–Hochberg false discovery rate (FDR) was used in order to filter out false-positive significant differences. Unweighted pair group method with arithmetic mean (UPGMA) with Bray–Curtis method was employed to cluster the samples and visualized by interactive Tree of Life (iTOL v.5.3) online platform (Letunic and Bork, 2019).

#### 2.3.2 Whole-Genome Sequence Analysis of Saliva Samples

Both read-based and genome-centric strategies were applied for the shotgun metagenome data analysis of saliva samples. Metagenomics data were analyzed with the workflow published in our previous study (Wirth et al., 2021). This contained the following main steps: 1) Raw sequence filtering: low-quality reads were filtered by Prinseq (Schmieder and Edwards, 2011) (default parameters, minlen: 100), and the quality of filtered sequences was checked with FastQC program. 2) Read-based metagenomics: filtered sequences were analyzed by Kraken2 (v.2.0.8) (confidence 0.8). Statistical analysis of read-based metagenomics data: this step is similar as previously described in amplicon sequence analysis module 5. 3) Genome-based evaluation of the sequencing data (Binning): filtered sequences were co-assembled to contigs with Megahit (Li et al., 2015) (min-contig-len 1,500). The original (filtered) sequences were mapped back to the contigs with Bowtie2 (v.2.3.4: –no-unal) (Langmead and Salzberg, 2012). For genome-based analysis, Anvi’o (v.5.3 “Margaret”) was used in the “metagenomics” workflow (Eren et al., 2015). Three automated binning programs, i.e., CONCOCT (v.1.1.0), METABAT2 (v.2.12.1), and MAXBIN2 (v.2.2.7), were employed to reconstruct microbial genomes from the contigs (all in default param.) (Alneberg et al., 2014; Kang et al., 2015; Wu et al., 2015). Anvi’o human guided binning was also employed based on the automated binning results. The results are further improved with “anvi-refine” option (Delmont and Eren, 2018). The Anvi’o interactive interface was used to visualize and summarize the data.

## 3 Results

### 3.1 Characterization of the Study Group

Nine periodontitis patients representing both genders with a mean age of 50.3 (40–62) years prior to non-surgical periodontal treatment were included in the clinical study. The subjects did not take antibiotics for the last 6 months, did not suffer from a known systemic illness, were non-smokers, and declared to have no drinking habit.

### 3.2 Sampling Methods and Microbial Communities

**Figure 1:**
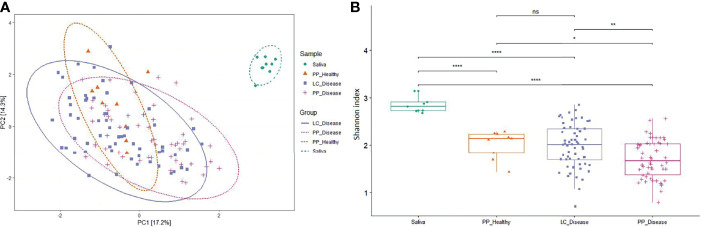
Principal coordinate analysis (PCoA) and alpha diversity. **(A)** The PCoA PC1 and PC2 dimensions represent 31.5% of the microbiome variation between amplicon [paper point (PP) and Langer curette (LC) samples] and metagenome sequencing (saliva) data. According to the PCoA, the sample groups are not significantly different (proven by PERMANOVA test, data not shown). **(B)** The alpha diversity of diseased samples shows a decreased tendency in microbial diversity. The differences in alpha diversity were calculated using the Wilcoxon test, which indicated a significant distinction between the sample groups; Cut points: 0–0.0001, 0.001, 0.05, 1; the corresponding symbols: ****, **, *, ns (not significant).

The Shannon index values, which account for both abundance and evenness of a population, were lower in the case of paper-point sampling than that of curette scaling samples, suggesting higher richness and evenness in the latter ones (**Figure 1**). The saliva samples showed the highest Shannon diversity index, which can be interpreted by taking into account the higher diversity in the planktonic oral microbiota relative to the sessile biofilms. Although the differences in the Shannon index values between the sample groups were significant, but due to the numerous outlier values, they reflect largely tendencies. The overlapping nature of the microbiomes is clearly demonstrated in the principal coordinate analysis (PCoA) plot (**Figure 1A**). Evaluation of the data with PERMANOVA gave essentially the same result (data not shown). The saliva samples were clearly distinct from any of the periodontal ones.

#### 3.2.1 The Core and the Most Abundant Microbes

**Figure 2:**
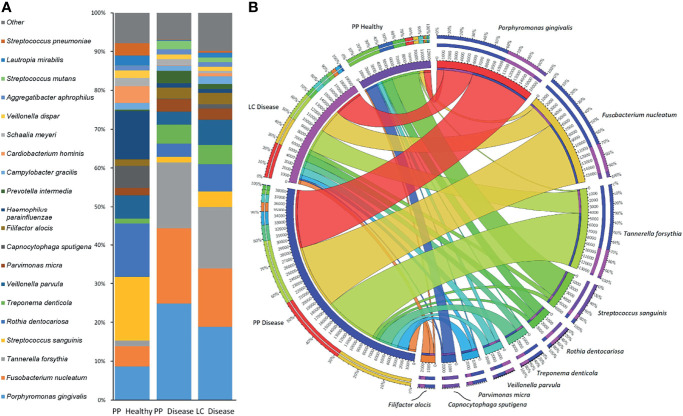
The most frequent microbes identified by amplicon sequencing. **(A)** The top 20 and **(B)** the top 10 microbes and their distribution between sample groups [paper point (PP) and Langer curette (LC) samples]. The *Porphyromonas–Fusobacterium–Tannerella* triumvirate dominates the periodontitis samples.

Since the sample groups, i.e., paper-point samples collected from clinically healthy teeth, paper-point samples collected from teeth affected by periodontitis, and curette samples collected from teeth affected by periodontitis, were not significantly different according to PCoA and Shannon diversity, the three metagenome datasets were treated together, and the resulting microbiome depicting the diseased and clinically healthy oral microbiome of the nine patients was compiled (**Figure 2**). **Figure 2A** presents the distribution of the top 20 most abundant bacterial strains in the three sample groups as identified using the RefSeq database. A salient difference between the clinically healthy and diseased microbiomes was apparent even upon superficial inspection. *Porphyromonas gingivalis* and *Tannerella forsythia*, two members of the “red complex,” characterized in subgingival plaque by Socransky et al. (2013) and *Fusobacterium nucleatum*, belonging in the “orange complex,” comprised about 15% of the total abundance in the clinically healthy sulcus, but they made up to 50%–60% of the community in the diseased periodontal pockets. The substantial decrease in the representation of the strains *Streptococcus sanguinis*, *Rothia dentocariosa*, *Veillonella parvula*, *Capnocytophaga sputigena*, and *Prevotella intermedia* in the periodontitis pockets relative to the healthy sulcus, together with increased abundances of *Treponema denticola*, *Parvimonas micra*, and *Filifactor alocis*, was indicative of severe inflammation. The 10 “most abundant” species represented the bulk of the detectable oral biofilm community (**Figure 2B**). More than half of the biofilm microbiomes were made up of the three top periodontopathogenic strains *P. gingivalis*, *F. nucleatum*, and *T. forsythia*. In spite of the perceivable differences, several abundant periodontopathogens, e.g., the genera *Treponema*, *Campylobacter*, *Filifactor*, were found in similar relative abundances following sampling by either the curette or paper-point method. Not very surprisingly, *S. sanguinis* and *C. sputigena*, which are primary colonizers and commensal oral bacteria, were predominant in the clinically healthy sulcus microbiome. *F. alocis* presented itself at the lowest prevalence among the “most abundant 10.” It has been recognized as a periodontal pathogen (Aruni et al., 2015) and has been detected in all three sample groups, although *F. alocis* was substantially more abundant in the two dysbiotic groups than in the gingival sulcus around the clinically healthy teeth (**Figure 2**).

#### 3.2.2 Differences Among the Sample Groups

Rigorous inspection, i.e., pairwise comparisons of the clinically healthy and periodontitis samples showed notable differences between these groups. The possible interference of using various publicly available and commonly used databases, i.e., the genome-based NCBI RefSeq (Reference Sequence Database, https://www.ncbi.nlm.nih.gov/refseq/) and the 16S rDNA amplicon-based RDP (Ribosomal Data Project, https://rdp.cme.msu.edu/), was also tested in this assessment. It is important to note that although the abundance patterns showed some differences, similar patterns emerged by using either the RDP or the RefSeq database, indicating that systematic bioinformatics bias was avoided (**Supplementary Material 4**).

The genus *Prevotella* embraces a diverse bacterial community. In our study, this was indicated by the uneven distribution of the three *Prevotella* species between the clinically healthy control and diseased samples. Although *P. melaninogenica* was the prevalent species from this genus, which was present primarily in the healthy control samples, *Prevotella* appeared to belong in the periodontal pathogen microbiome group at the genus level (see also the *Saliva Microbiota* section below).

*Tannerella* was represented by a single strain, *T. forsythia*, beyond threshold, contrasting the diverse genus *Prevotella*, but showed a huge relative abundance increase from <1% in the clinically healthy control samples to 16%–17% in the diseased periodontal pockets.

### 3.3 Distinct Periodontopathogenic Microbial Clusters

**Figure 3:**
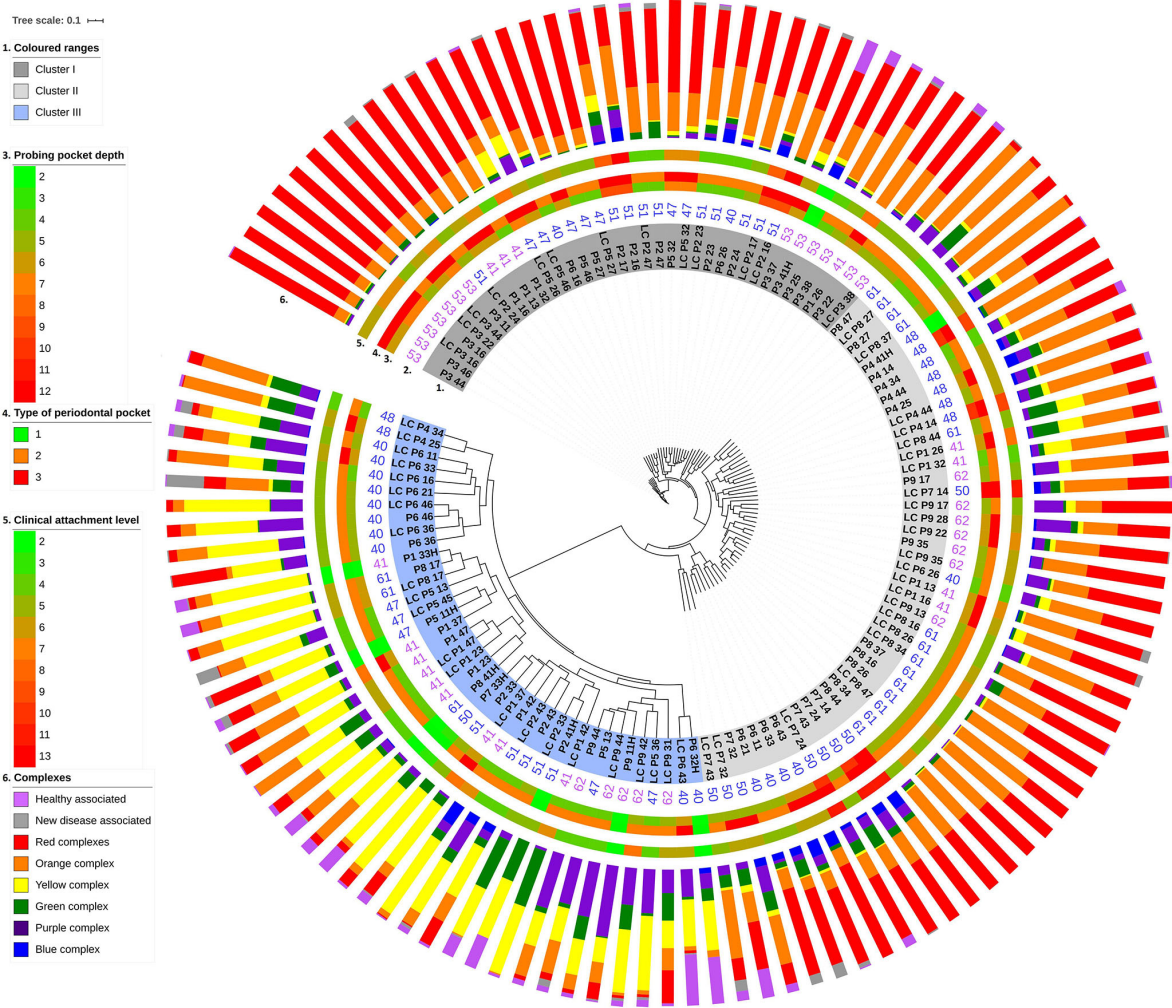
Unweighted pair group method with arithmetic mean (UPGMA) cluster analysis of supragingival biofilm metagenomes. The study group designation (capital letters), the numbers marking the anonymous individual patients and sampled tooth positions are highlighted in dark gray, light gray, and light blue backgrounds according to their position on the major branches of the UPGMA tree (innermost ring #1). Ring #2: Patient’s age. Rings #3–5: Probing pocket depth (PPD), PPD type, and clinical attachment level (CAL). Scales for these parameters are indicated on the left side. Ring #6: Composition of microbial communities according to the Socransky complexes (see **Figure 4**).

A detailed look at the biofilm taxonomy distributions and associated metagenomes disclosed three clearly distinguished clusters (**Figure 3**). The tree split very strongly into two clusters centered in the middle and the third one around the distant end of the tree. The metagenomes associated with the distant cluster are marked with blue background color in the innermost ring #1. The other two, highlighted in dark and light gray colors, formed separate clusters indicated by the distinct lengths of the cluster tree branches. These microbiome clustering patterns did not correlate with any of the following parameters: sampling method, i.e., paper-point or curette, the position of the sampled tooth (**Figure 3**, ring #1), and age and gender of the patients (**Figure 3**, ring #2). Additional parameters tested for correlation with the microbiome clustering were as follows: Gingival Index, number of tooth roots, and Plaque Index (data not shown). None of these showed perceivable correlation with the clusters. Systemic illness, smoking, and drinking habits have been excluded in the selection of the subjects (See section *Characterization of the Study Group*). Conversely, a good relationship was recognized between three interrelated parameters and the microbiome clustering. These parameters were as follows: probing pocket depth (**Figure 3**, ring #3), type of periodontal pocket (**Figure 3**, ring #4), and CAL (**Figure 3**, ring #5). Taken together, these observations suggested the formation of more than one well-defined, apparently stable “diseased” microbiota patterns in the biofilms of periodontitis subjects.

#### 3.3.1 The Composition of Microbiome Clusters

**Figure 4:**
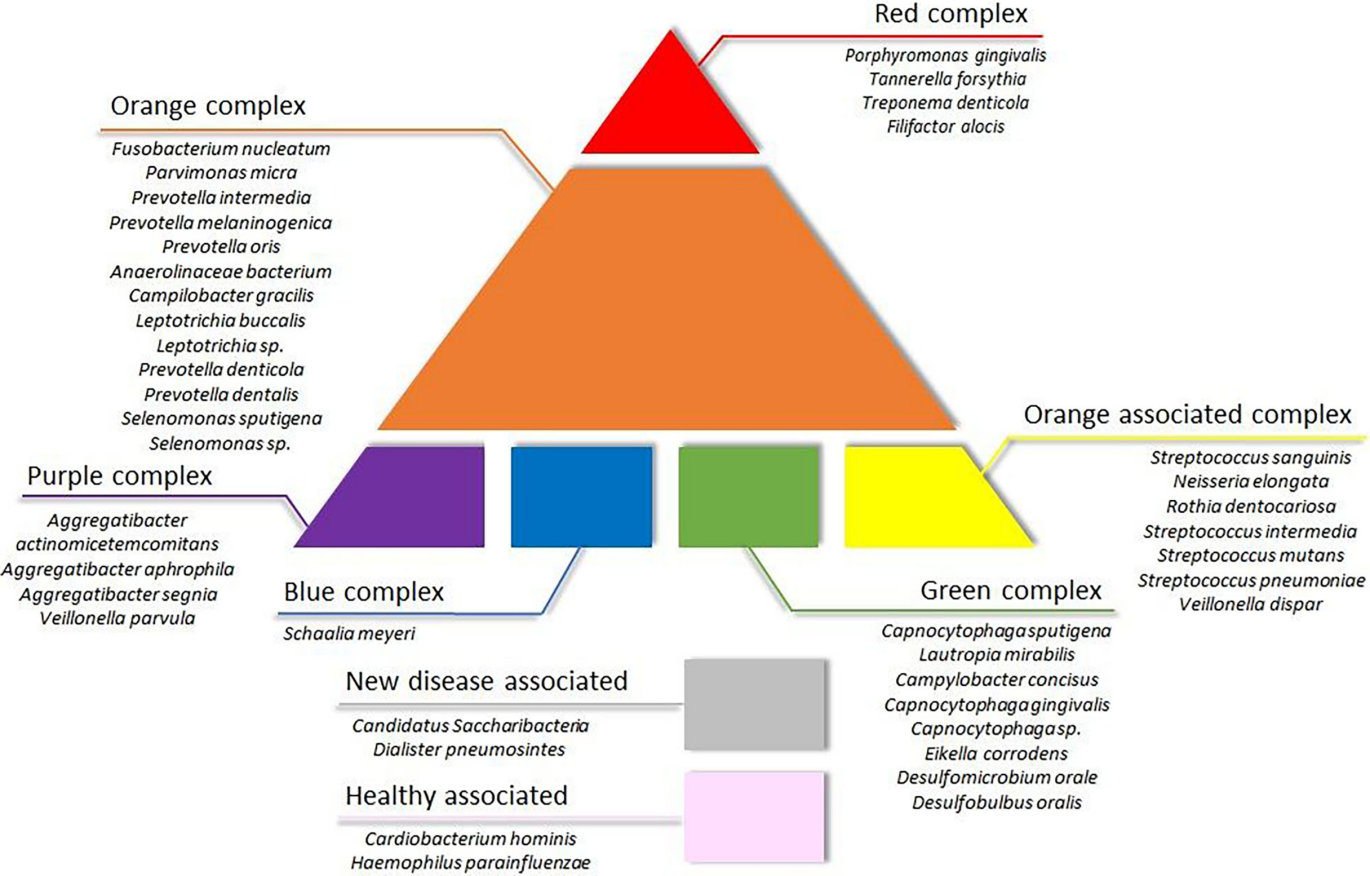
Updated scheme of oral microbial complexes. Color coding of the bacterial groups was based on their association with microbial complexes involved in oral pathogenesis, indicating their potential contribution to oral health (Socransky et al., 1998; Haffajee et al., 2008; Colombo and Tanner, 2019).

**Figure 5:**
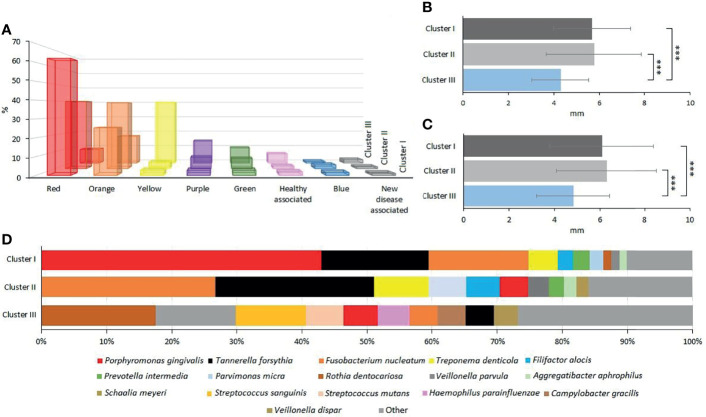
Evaluation of oral microbial clusters. **(A)** The percentage distribution of microbial complexes between the clusters. **(B, C)** The average of the clusters’ probing pocket depth (PPD) and clinical attachment level (CAL), respectively. **(D)** The percentage abundances of predominant species in Clusters I, II, and III, respectively.

The next task was the identification of distinct and common elements of the three identified clusters. In this analysis, the classification of the oral microbial taxa in microbial complexes (**Figure 4**) (Socransky and Haffajee, 2005; Socransky et al., 2013; Kumar et al., 2020) was followed with amendments suggested by this and earlier studies. Notably, we propose to place *F. alocis* in the group of “red” complex periodontopathogens (Al-hebshi et al., 2015; Schulz et al., 2019; Lee et al., 2020; Aja et al., 2021) and added new species to the bottom boxes of the pyramid. Interesting differences could be recognized according to the distribution of microbial complexes (**Figures 5A, D**). The “red complex,” which comprises the most pathogenic bacteria, was predominant in Cluster I, while the commensal “yellow,” “purple,” “green,” and “healthy associated” complexes were more abundant in Cluster III (**Figure 5D**). Cluster II was in the middle of the two boundaries; its prevalent complex was the “orange” one, which is usually ranked as a less potent periodontal pathogen relative to the “red” complex (**Figure 5A**). Based on this distribution of microbiome complexes, one could assign Cluster I to be the most periodontopathogenic microbial pattern and Cluster III to be the least periodontopathogenic microbial community. Cluster I and Cluster II displayed characteristically distinct microbial patterns (**Figure 5D**), which were predominated by *P. gingivalis* in Cluster I and *F. nucleatum* in Cluster II. In both clusters, *T. forsythia* was the second most abundant bacterium. It is noteworthy that the diversity of the microbiomes increased from Cluster I toward Cluster III.

**Figure 6:**
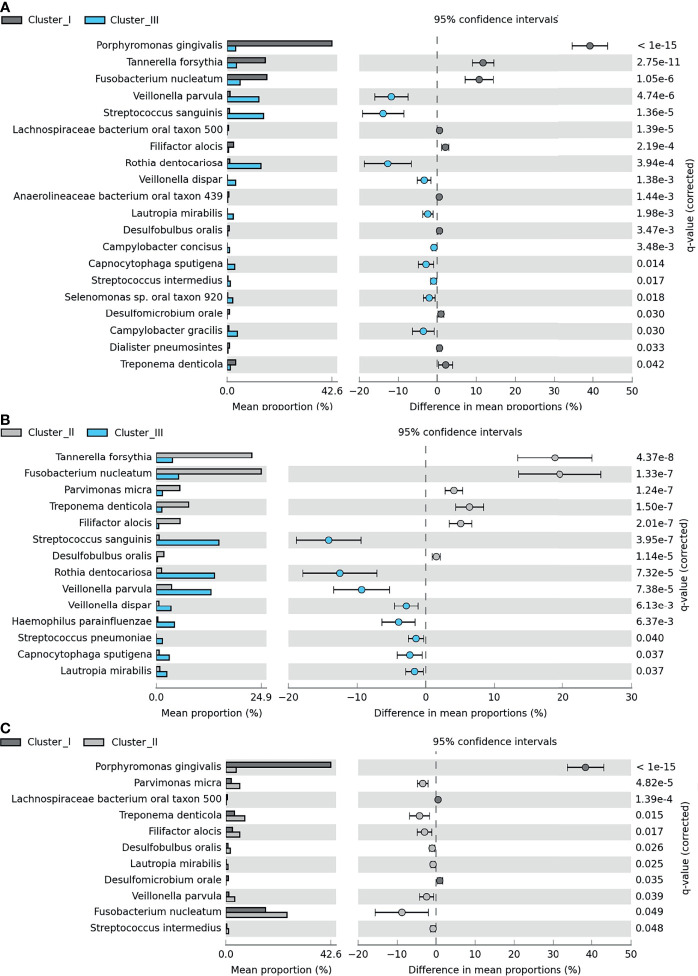
Pairwise differences among Clusters I–III. Significant differences between **(A)** Cluster I (dark gray) and Cluster III (light blue), **(B)** Cluster II (light gray) and Cluster III, and **(C)** Cluster I and Cluster III. The bar charts represent the mean proportion (%) of specific microbial species in a specific cluster, and the plots show the differences in mean proportions (%). For calculation, see *Materials and Methods* section *Amplicon Sequence Analysis of Subgingival Biofilm Samples*.

Clustering, based on the distribution of microbiome complexes, apparently correlated with probing pocket depth (PPD) (**Figure 5B**) and CAL (**Figure 5C**). Both PPD and CAL values of Cluster I and Cluster II samples significantly differed from those of Cluster III. In case of PPD, the difference between Cluster I and Cluster III was significant at p = 0.0009, while between Cluster II and Cluster III, it was significant at p = 0.0002. For CAL, the same significance levels were p = 0.0066 and p = 0.0009, respectively. The bacteria present in significantly different (p ≤ 0.05) abundances (**Figure 6**) showed a clear trend ranging from microbiomes rich in Cluster I components to microbiomes predominated by Cluster III microbes and a few microbiomes falling in between, i.e., displaying mixed Cluster I and Cluster II microbes.

**Figure 7:**
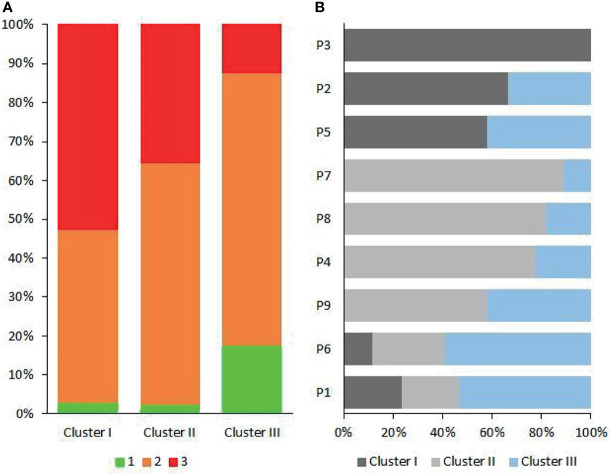
The patient-dependent distribution of clusters. **(A)** The contribution of probing pocket depth types (PPDTs) in the three oral microbial clusters. **(B)** The distribution of Clusters I–III in individual patients (P1–P9).

In dental practice, periodontitis pockets are frequently classified in three categories of probing pocket depth types (PPDT) 1–3 (see *Materials and Methods*). The Cluster I microbiomes (**Figures 3**, **5D**) were found primarily in the PPDT-3-type pockets; a prime example was found in Patient-3 as all of his/her periodontal pocket samples exhibited Cluster I-type microbiome complex distribution including the clinically healthy control sulcus sample (P341H on **Figure 3**). Cluster I-type pattern of microbiome complexes could therefore be classified as characteristic of “severe periodontitis” condition. Cluster III-type microbiomes characterized the least severe diseased pockets (**Figure 5A**). Although several “diseased” periodontitis biofilm samples showed microbial complex composition characteristic of Cluster III, 10 microbiomes out of the 12 samples taken from Patient-6 also mapped into this cluster, suggesting a less severe or less advanced periodontitis in this case. It is also important to note that seven of the total of nine control healthy teeth biofilm samples also displayed Cluster III-type distribution of microbiome complexes (**Figure 3**). The clinically healthy samples did not disturb the overall “periodontitis” cluster composition; removing these nine samples would result essentially in the same cluster composition of the remaining 112 individual samples. Cluster II microbiomes occupied a position in between Cluster I and Cluster III, and they were characterized mostly by PPDT-2 pocket types. This indicated a rather strong correlation and distinction based on microbiome complex distribution and PPDT diseased pocket typing (**Figure 7A**) and further established the pathogenic implications of more than one cluster, based on microbiome complexes, in the periodontitis biofilm oral community (**Figure 3**). Taken together, there is a clear indication for the existence of more than one well-defined “pathogenic periodontitis microbiome pattern” if one looks at the distribution of microbial complexes at higher resolution than most of the previous studies did.

### 3.4 Microbiome Clusters in the Individual Patients

The clusters, distinguished on the basis of microbes in the individual clinically healthy and periodontitis biofilms, could be projected back to the individual patients’ (N = 9) microbiomes. From each patient, samples were collected from 4–8 periodontal pockets. In addition, a control microbial sample was also taken from each subject, using a paper point, from the crevicular fluid of a single clinically healthy gingival sulcus. The proportions of the Probing Pocket Depth Types (PPDT: 1-green, 2-orange, 3-red) in Clusters I–III are shown in **Figure 7A**, indicating the strong correlation between PPDT and Cluster I–III microbiomes. The number of samples displaying features characteristic of Clusters I–III were counted, and the fractions of Clusters I–III are plotted for each patient in **Figure 7B**. This clearly showed that the distribution of Clusters I–III was patient dependent (**Supplementary Material 4**). Patient-3 and Patient-6 displayed the two extremely distinct microbiomes. This implicated that once a predominantly periodontopathogenic microbiota established itself in a person’s oral cavity, it remained there for a long time, and its composition was unlikely to change randomly or systematically (Buduneli, 2021; Feres et al., 2021).

### 3.5 Saliva Microbiota

**Figure 8:**
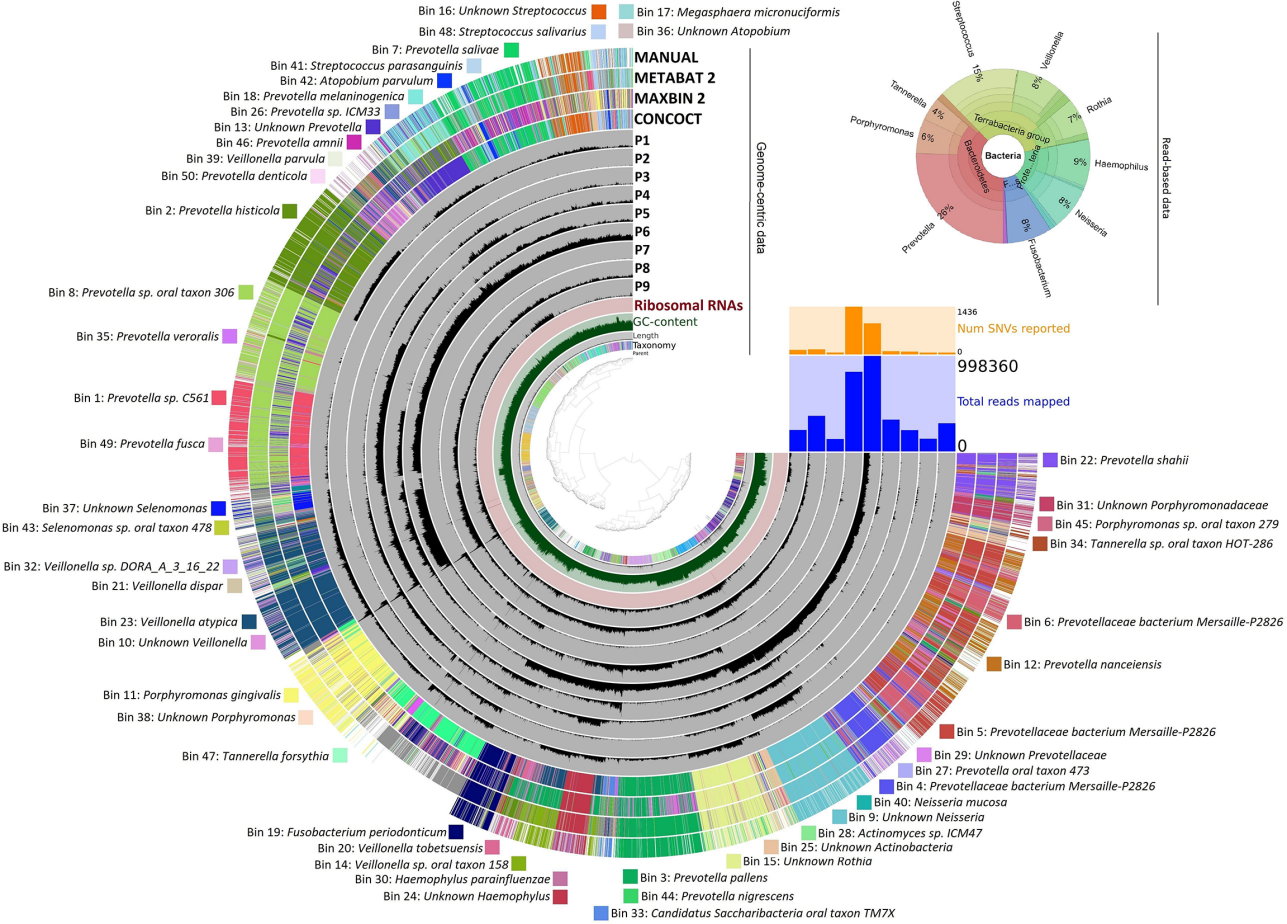
Bacterial taxa identified in saliva samples. Genome-centric data: The distribution of contigs is plotted in the rings. The grouping of contigs was based on sequence assignments of automated binning programs METABAT2, MAXBIN2, and CONCOCT, as well as manually defined bins as presented on the Anvi’o platform. The list of identified bins is given around the figure. Read-based data represent the relative abundance of predominant genera.

Whole-genome deep sequencing of the total saliva DNA made possible both read-based and genome-based, i.e., binning, evaluation of the data (Pasolli et al., 2019; Wirth et al., 2020; Zaura et al., 2021). The results are summarized in **Figure 8**.

In the upper right corner, the read-based whole-genome sequencing results are summarized (The detailed data are compiled in **Supplementary Material 4**). The Krona representation of the taxa at genus resolution indicated several features, which were distinct from the similar distribution profile gained in amplicon sequencing (**Figure 8**). There was a major distinction in the abundance of the genus *Prevotella*, which was the most predominant taxon in the saliva. *Prevotella* was detected in the amplicon sequencing of the periodontal pockets’ biofilms but with much lower abundance (<3%). Surprisingly, the genus *Prevotella* predominated the saliva microbiota by being the single genus representing 26% of the read-based metagenomics sequences. The massive difference in *Prevotella* abundances between the saliva and periodontal pockets suggests that *Prevotella* may be particularly susceptible to shedding from the biofilms in periodontitis patients. The significance of *Prevotella* implication in periodontitis was even more apparent from the genome-based binning results. Here, 50 metagenome-assembled genomes (MAGs) could be identified and assigned to various microbial species or taxa (**Figure 8**). Out of the positively identified 50 bins, 19 belonged to individual *Prevotella* strains. This indicated a highly diverse distribution of *Prevotella* strains in the saliva of the periodontal patients.

## 4 Discussion

### 4.1 Sampling Methods

Several methods have been described in the literature to effectively remove microbial samples from the soft and hard surface-attached biofilms using swabs, paper points, or curettes (Beikler et al., 2006; Persson et al., 2008; Teles et al., 2008; Belibasakis et al., 2014; Santigli et al., 2017; Zaura et al., 2021). In this study, the microbiomes after sampling the same individual periodontal pockets using paper points and LCs were investigated. The two sets of microbiomes were not identical, which warrants the implementation and use of a standardized protocol for sampling, particularly when various data sets are to be compared. This demonstrated that correct pictures about the composition of the key pathogens in the diseased biofilms can be drawn by these sampling methods, but standardization of the sampling techniques is warranted in order to make various experiments comparable.

Essentially the same group of the most abundant 10 or 20 species were found in the three sample groups, i.e., clinically healthy gingival sulcus samples and samples from periodontal pockets collected with paper point and curette (**Figures 1**, **2**). Nevertheless, members of the “red” and “orange” microbial complexes (**Figure 4**) were more abundant in the diseased biofilms relative to the clinically healthy controls (Feres et al., 2021; Kumar et al., 2021). It is noteworthy that the sampling strategy employed in this study, i.e., handling the samples taken from the individual sites separately, avoided the frequently encountered flaws instigated by pooling biofilm samples or considering the individual as the unit of analysis. This approach is more laborious and costly but allows substantially increased resolution. For example, in one of the recent exhaustive related studies (Dabdoub et al., 2016), the “healthy,” “shallow-diseased,” and “deep-diseased” endodontic paper-point samples were pooled and subjected to whole-genome sequencing (WGS). Pooling was necessary because of the high proportion (63%–77%) of human sequences obtained in the WGS sequences, albeit the extra functional information offered by the WGS strategy of “shallow” and “deep” periodontal pockets’ microbiomes.

An entirely different partition of the microbiome datasets was recognized when all 121 microbiomes were mapped according to their microbial composition (**Figures 3**, **5**, **6**). This analysis revealed a distinct clustering. In two of them, members of the “red” and “orange” microbial complexes predominated, although in very different representations. Although both *F. nucleatum* and *T. forsythia* were predominant in both clusters, their abundances were markedly different (**Figures 5A, D** **)**. The microbial composition of Cluster III was outstandingly distinct from those of Cluster I and Cluster II, and the microbial diversity increased from Cluster I to Cluster III (Kumar et al., 2021). Several clinical and diagnostic parameters were tested, but no correlation with the Cluster grouping was found, except for PPDT and CAL. These observations suggest that distinct periodontal pathogenic microbiomes can develop and survive around the diseased teeth within the oral cavity of the individual patients diagnosed with the same disease. This is likely due to the rarely recognized “personalized microbiota” phenomenon (Zaura et al., 2017; Scannapieco and Dongari-Bagtzoglou, 2021). Moreover, in a few cases, distinct microbiomes were found (P6 and P1 in **Figure 7B**) in the oral cavity of the same patient. This can explain the variability of the clinical results when the patient responds poorly to the therapy in a few sites while presents good clinical response in others. Hence, therapeutic intervention aiming at a single or a limited number of periodontal pathogen(s) may not be the most efficient approach to treat all periodontitis patients. In future studies and personalized medicine practice, larger cohorts of patients and more extended physiological/immunological characterization of the subjects will be necessary to disclose the precise mechanisms of interactions between the individual patient and his/her microbiota (Cugini et al., 2021; Kumar et al., 2021; Teles et al., 2021).

### 4.2 Saliva Microbiomes

Saliva is considered as a valuable diagnostic fluid (Malamud and Rodriguez-Chavez, 2011; Shet et al., 2013; Baima et al., 2021) to search for various biomarkers establishing the oral health status. The comparison of the microbiomes in the periodontal subgingival biofilms and saliva of the same subjects offered a novel dimension to understand the microbial communities persistent in the oral cavity of periodontitis patients. By its nature, saliva can give only a fuzzy picture of the oral microbiome; therefore, it is not suitable for mapping the differences among periodontal sites. Nevertheless, the major and most abundant periodontal pathogens detected in the subgingival biofilms were also present in the saliva, although the abundances of the various genera were different from those found in the biofilm samples. A notable dissimilarity was the 6–8-fold abundance increase of the genus *Prevotella* in saliva relative to the <3% representation in the diseased biofilms. Apparently, the genus *Prevotella* could be considered as an indicator taxon of oral inflammation in saliva. Indicator strain-specific resolution could not be recommended because of the diversity of *Prevotella* strains in the saliva, but it is noteworthy that the genus *Prevotella* was detected in similarly high predominance, i.e., 24%, in the saliva of adolescent gingivitis patients (Wirth et al., 2021), whereas their representation in the plaques’ microbial community was low. These findings may suggest the genus *Prevotella* as salivary indicator microbial taxon of oral inflammation in general.

It is unclear why *Prevotella*, a biofilm-forming microbe, apparently accumulates in the saliva relative to the biofilms of patients suffering in various degrees of oral inflammation. This phenomenon is perhaps a result of host–microbiota interaction regulated by biofilm deconstructing antimicrobial proteins produced by the host (Wakabayashi et al., 2009). Alternatively, the wide diversity of microbial strains belonging in the genus *Prevotella* as revealed in the genome-centric evaluation of saliva data (**Figure 8**) may explain the high abundance of certain *Prevotella* strains in the saliva.

The genus *Tannerella* was represented by the single strain *T. forsythia* in both the periodontal pockets and saliva. The genus *Tannerella* appeared in the read-based whole-genome saliva analysis with 4% abundance. This was higher than *Tannerella* abundance in the healthy or gingivitis saliva (Wirth et al., 2021) but not as marked as the difference between “clinically healthy” sulcus (about 1%) and periodontal pocket whole-genome representation (16%–17%). Since *T. forsythia* was found in the diseased pockets in about 16–17-fold excess relative to the clinically healthy sulcus, it may become an excellent indicator strain for pocket-bound periodontitis biofilms.

Overall, our findings corroborated the importance and diagnostic value of the *Porphyromonas–Fusobacterium–Tannerella* triumvirate, indicating the potential importance of the “specific plaque hypothesis” (Pasolli et al., 2019; Wirth et al., 2020; Buduneli, 2021) and the “non-specific plaque hypothesis” (Cugini et al., 2021) in describing the oral microbiota. *Tannerella* alone may be considered as an indicator taxon in the periodontal pocket samples, whereas *Prevotella* seems to be a more prominent indicator genus of the disease in the saliva.

Apart from the potential diagnostic values, a more detailed and comprehensive description of the various versions of the oral microbial “communities as pathogens” and “personalized pathology” (Feres et al., 2021) adds new components to the knowledge about the microbial background of periodontitis, a multifactorial and complex dysbiotic status between the microbiota and host. The ultimate goal, i.e., personalized therapy, which may involve selective antibiotics and/or probiotics designed by taking into account the patient’s health and immune status, is far away (Feres et al., 2021). The design of precisely targeted personalized therapy is complicated by the fact that different bacterial clusters may be present in the same person. The strategy of sampling and analyzing the diseased sites individually is a prerequisite for developing accurate personalized therapy. This is a feasible strategy in the case of chronic periodontitis, where several visits of the patients to the dentist are required and the few weeks in between the treatments are plenty enough to process the samples and do the sequencing and the bioinformatics evaluation.

## Data Availability Statement

The datasets presented in this study can be found in online repositories. The names of the repository/repositories and accession number(s) can be found below: https://www.ncbi.nlm.nih.gov/, PRJNA682523.

## Author Contributions

RW: data curation and evaluation, supervision, and writing—review and editing. BP: formal analysis, investigation, and methodology. GM: conceptualization, methodology, supervision, funding acquisition, and writing—original draft. PV: conceptualization and sample collection. LK: formal analysis, investigation, and methodology. NB: data curation, investigation, and methodology. OS: data curation, investigation, methodology, and writing—original draft. JM: conceptualization, project administration, supervision, and writing—original draft. KK: conceptualization, methodology, supervision, project administration, and writing. All authors contributed to the article and approved the submitted version.

## Funding

This work was supported by the European Regional Development Fund to a project led by JM (grant no.: GINOP-2.3.2-15-2016-00011). RW (PD132145) and GM (FK123899) received support from the National Research, Development and Innovation Office (NKFIH), Hungary.

## Conflict of Interest

The authors declare that the research was conducted in the absence of any commercial or financial relationships that could be construed as a potential conflict of interest.

## Publisher’s Note

All claims expressed in this article are solely those of the authors and do not necessarily represent those of their affiliated organizations, or those of the publisher, the editors and the reviewers. Any product that may be evaluated in this article, or claim that may be made by its manufacturer, is not guaranteed or endorsed by the publisher.

## Supplementary Material

Supplementary Material 1 Participants’ data. Columns list the data as follows: **(A)** Sample names indicate the individual subjects’ code and the tooth position in the quadrat. H stands for “clinically healthy” tooth. **(B)** age of the participant. **(C)** gender of the participant (male/female). **(D)** Sampling sites: db, disto buccal; dl, disto lingual; dp, disto palatinal; ml, mesio lingual; mp, mesio palatinal; mb, mesio buccal. **(E)** type of the pocket on the scale of 1-3. **(F)** roots: MR, multiple root; SR, single root. **(G)** Probing pocket depth. **(H)** Clinical attachment level (CAL), **(I)** gingival index (GI), **(J)** plaque index (PI).

Supplementary Material 2 Sample ID table.

Supplementary Material 3 STAMP data.

Supplementary Material 4 Amplicon sequencing data. Page 1: RefSeq data, Page 2: RDP data. In the header the numbers mark the anonymous individual patients and sampled tooth positions (LC and H mean Langer curette and healthy control samples, respectively).
